# Diagnostic and Clinical Utility of Novel Anti-PAD4 Antibodies in Anti-CCP2-Negative and Anti-CCP2-Positive Rheumatoid Arthritis

**DOI:** 10.3390/diagnostics16213615

**Published:** 2026-11-01

**Authors:** Tyler O’Malley, Brittany Partain, Cynthia Alvarez, Clayton Nagle, Michael Mahler

**Affiliations:** Exagen, Inc., Vista, CA 92081, USA

**Keywords:** rheumatoid arthritis, anti-PAD4, autoantibodies, seronegative, anti-CCP2, biomarkers, diagnostic performance

## Abstract

**Background/Objectives:** Diagnosis of rheumatoid arthritis (RA) remains challenging, particularly in anti-CCP2-negative disease. Novel biomarkers, including anti-peptidyl arginine deiminase 4 (PAD4) autoantibodies, have emerged as promising tools for improving diagnostic accuracy in RA patients. The aim of this study was to analyze the performance of the first clinically validated anti-PAD4 assay. **Methods:** A total of 192 well-characterized RA patients and 383 controls were included in this study and tested for anti-CCP2, RF IgA, and RF IgM (all Phadia, Thermo Fisher) as well as anti-PAD4 IgA and anti-PAD4 IgG by ELISA (laboratory developed test, Exagen, Inc., Vista, CA, USA). Results for the anti-PAD4 IgG ELISA for 143 patients were used for method comparison with a particle-based multi-analyte technology (PMAT, Inova Diagnostics, San Diego, CA, USA). **Results**: ELISA and PMAT showed moderate-to-good agreement for anti-PAD4 IgG (rho = 0.66; Passing–Bablok slope 0.83, 95% CI 0.71–0.92; kappa = 0.67). Anti-PAD4 IgG was significantly correlated with anti-PAD4 IgA (rho = 0.47) and weakly correlated with RF IgM (rho = 0.29), RF IgA (rho = 0.28), and anti-CCP2 (rho = 0.22) (all *p* < 0.001). The PAD4 IgG and IgA ELISAs were validated in the RA population, yielding sensitivities/specificities of 31.8%/92.7% for anti-PAD4 IgG, 18.8%/93.7% for anti-PAD4 IgA, 43.2%/91.1% for RF IgM, 23.4%/96.1% for RF IgA, and 42.2%/99.2% for anti-CCP2, respectively. Anti-CCP2 IgG achieved the highest discriminatory performance (AUC = 0.86), followed by RF IgA (AUC = 0.69) and RF IgM (AUC = 0.67). Anti-PAD4 IgG and anti-PAD4 IgA showed modest discrimination (AUC = 0.55 and 0.52, respectively). Among the biomarkers evaluated, anti-CCP2 demonstrated the strongest association with RA (OR = 110.27), followed by RF IgM (OR = 7.40), RF IgA (OR = 7.14), anti-PAD4 IgG (OR = 5.92), and anti-PAD4 IgA (OR = 3.48) (all q < 0.001). In the anti-CCP2-negative population, the strongest association was found for anti-PAD4 IgG (OR = 2.87, q = 0.005), followed by RF IgM (OR = 2.20, *p* = 0.024). After adjustment for anti-CCP2, RF, age, and sex, anti-PAD4 IgG remained independently associated with RA (OR = 2.69, *p* = 0.003), with a small gain in discrimination (ΔAUC = 0.02–0.04). **Conclusions**: Our study established comparability between methods and shows that anti-PAD4 provides modest but statistically significant diagnostic information in the anti-CCP2-negative RA population, independent of anti-CCP2 and RF, in the anti-CCP2-negative RA population, where conventional markers are uninformative.

## 1. Introduction

Rheumatoid arthritis (RA) is a heterogeneous chronic systemic inflammatory disease affecting synovial joints and periarticular structures, which can lead to comorbid conditions affecting vascular, cardiac, skeletal, and neurologic structures [1]. Abnormalities in the conventional serologic biomarkers rheumatoid factor (RF) and anti-citrullinated peptide antibodies (ACPAs) are part of the 2010 American College of Rheumatology (ACR) classification criteria for RA [2]. While these biomarkers are highly valuable, a gap persists, especially in early disease [3]. Although seronegative RA has been defined in various ways, ACPA is the most disease-specific of the conventional markers and the principal contributor to this gap (20–30% of established RA test negative). Because a proportion of ACPA-negative patients remain RF-positive, we refer throughout to anti-CCP2-negative and anti-CCP2-positive RA rather than to seronegative and seropositive RA. The absence of ACPAs is significantly more common in early RA, with nearly 50% of patients testing negative for conventional biomarkers [3,4]. Thus, anti-CCP2-negative patients pose an immense challenge to achieving timely RA diagnosis.

RA diagnosis is often further complicated by nonspecific symptoms coupled with negative serologies. Symptom overlap between RA and other autoimmune rheumatic diseases (ARDs) and missing classical RA features can lead to misclassification of patients, delaying timely diagnosis and prompt intervention. These factors can prolong the time from initial joint swelling to fulfillment of ACR classification and initiation of disease-modifying antirheumatic drugs (DMARDs), especially in seronegative patients [5]. Timely diagnosis is imperative, as early intervention is associated with reduced disease burden, and RA-related morbidity can be exacerbated by diagnosis delays. A study by Rosa et al. [6] reported higher levels of structural damage, as evaluated by Sharp–van der Heijde scores, in patients with diagnosis delays greater than 12 months compared to those with shorter delays (*n* = 70; mean score of 30.9 vs. 21.3, respectively; *p* < 0.05). Further, patients who achieved diagnosis and initiated DMARD therapy three months after RA symptom onset showed reduced disease activity (DAS28) and slower progression than patients who initiated treatment roughly 12 months after symptom onset [7]. While serology is pivotal to RA diagnosis, the lack of reliable biomarkers in anti-CCP2-negative RA remains a critical gap that impedes timely diagnosis and subsequent intervention.

Several novel biomarkers have been described in RA, including, but not limited to, anti-RA33 antibodies (IgA, IgG, and IgM), anti-peptidyl arginine deiminase (PAD) antibodies, and 14-3-3η [3,8,9]. Anti-PAD4 antibodies (IgA and IgG) are directed against PAD4, which is involved in post-translational citrullination of protein residues, a hallmark of RA [10]. Anti-PAD4 has shown promise as an early serologic indicator for RA, as positivity often precedes diagnosis by 4–5 years [11,12], and it can be detected in both seropositive and seronegative patients [13]. Anti-PAD4 antibodies are specific for RA against healthy controls and can differentiate RA from other ARDs, including systemic lupus erythematosus (SLE), Sjögren’s disease (SjD), and psoriatic arthritis (PsA). Beyond diagnostic utility, anti-PAD4 positivity could inform RA prognosis related to disease severity and treatment response [14,15,16]. In established RA (disease duration ≥ 2 years), anti-PAD4 positivity at baseline was associated with more severe disease. Simultaneously, anti-PAD4 positivity was associated with a greater decline in DAS28 score post-treatment in patients with early RA (disease duration < 2 years) [15].

Recently, anti-PAD4 antibody testing became clinically available in the United States via a laboratory developed test (LDT). To translate research findings into clinical practice, comparison of immunoassays is important for estimating their comparability. Anti-PAD4 antibodies not only provide diagnostic utility but are also directly involved in disease pathogenesis via two independent mechanisms. First, a subpopulation of anti-PAD4 antibodies has been shown to mimic calcium binding, which leads to activation of the PAD4 enzyme and ultimately to enhanced citrullination [8,16,17,18]. Additionally, anti-PAD4 antibodies bind to PAD4 exposed on monocytes, which in turn triggers cytokine release and impacts fibroblasts [19]. These mechanisms provide a strong scientific rationale for assessing anti-PAD4 antibodies in patients with inflammatory arthritis.

The current study evaluated the diagnostic performance of anti-PAD4 antibodies, including stratification by individual isotypes, in anti-CCP2-negative and anti-CCP2-positive patients. Additionally, we compared particle-based multi-analyte technology (PMAT, Inova Diagnostics, San Diego, CA, USA) with the first clinically validated ELISA used for patient testing (LDT, Exagen, Inc., Vista, CA, USA).

## 2. Materials and Methods

### 2.1. Patients

Study participants were enrolled consecutively in a cross-sectional design across 18 clinical research sites in the United States between 2011 and 2025. Specimens were drawn from the MOSAIC protocol (Multi-center Observational Study of Autoimmune Immunologic Characterization; sponsor protocol 25-ARD1-MOSAIC), approved by WCG IRB (tracking number 20251884), and from previously concluded, bio-banked collections obtained under institutional IRB-approved protocols conducted in accordance with ICH guidelines. The cohort comprised 575 subjects: 192 RA patients meeting the 2010 ACR/EULAR classification criteria, 205 apparently healthy volunteers (AHVs), 79 SLE patients meeting the 1997 ACR criteria, 35 clinically diagnosed SjD patients, and 64 clinically diagnosed PsA patients. All participants were adults who provided written informed consent for themselves. Serum was collected at a single study visit, aliquoted, and stored at −80 °C at Exagen (Vista, CA, USA) until testing; for subjects with more than one sample, only the earliest visit was retained. Sex was available for all subjects, and age for 564 of 575 (missing for 2 RA patients and 9 controls); disease duration and disease stage were available for 189 of 192 RA patients, CDAI for 155, and treatment data for 132. No imputation was performed; age-adjusted models and analyses of clinical variables were restricted to subjects with complete data. Laboratory scientists were blinded to clinical data, and statistical analyses were performed by an independent biostatistics team.

### 2.2. Diagnostic Testing

Enzyme-linked immunoassay (ELIA) testing was performed to measure anti-cyclic citrullinated peptide 2 (CCP2) IgG and RF (IgA and IgM) all on the Phadia 2500 instrument (Thermo Fisher, Freiburg, Germany). Anti-PAD4 antibodies (RUO, IgA and IgG) were measured by ELISA (provided by Werfen/Inova Diagnostics; San Diego, CA, USA; Research use only provided under a research grant). Cutoffs for anti-CCP2 and RF were based on the manufacturer’s specifications or as previously established. Anti-PAD4 antibody cutoffs for each isotype were set above the 95th percentile for AHVs. RA subjects were stratified by anti-CCP2 status for all subgroup analyses; because some anti-CCP2-negative patients were RF-positive, this group is referred to as anti-CCP2-negative rather than seronegative. A total of 143 samples, that had sufficient sample volume available were tested for anti-PAD4 (IgG and IgA) by ELISA and compared to historical data [13] generated using PMAT. This study used an early version of the anti-PAD4 IgG assay and a prototype Aptiva instrument.

### 2.3. Statistical Methods

Demographic and clinical characteristics were summarized by subgroup (RA, AHV, SLE, PsA, and SjD) and by RA subgroup (anti-CCP2 positive and anti-CCP2 negative) using descriptive statistics; categorical variables were reported as counts and percentages, while continuous variables were reported as means with standard deviations or medians with interquartile ranges (IQRs), as applicable. Comparisons between the RA group and all controls combined were made using the Mann–Whitney U test and Pearson’s Chi-square test or Fisher’s exact test, as appropriate. Comparisons between the RA subgroups were made using Pearson’s Chi-square test and the Mann–Whitney U test.

Anti-PAD4 IgG measured by ELISA and PMAT was compared in 143 of the 575 samples: those with sufficient residual volume for which PMAT results were available (132 RA, 9 PsA, 2 AHV; 57 anti-CCP2 positive, 86 negative). Positivity cutoffs were ≥13 U/mL (ELISA) and ≥1000 MFI (PMAT). Rank correlation was assessed by Spearman’s rho. Quantitative agreement was assessed on log10-transformed values, using Passing–Bablok regression (primary) and Deming regression with error ratio 1 (sensitivity), with 95% bootstrap CIs; residuals were inspected for non-linearity. Samples with ELISA values > 2 SD above the Passing–Bablok prediction were flagged, and the regression was repeated without them. Qualitative agreement was assessed using positive, negative and overall percent agreement (PPA, NPA, POA; PMAT as comparator; Wilson 95% CIs), Cohen’s kappa (Fleiss–Cohen–Everitt CIs), and exact and continuity-corrected McNemar tests. A PMAT cutoff maximizing agreement with the ELISA call was derived by Youden’s index. All analyses were repeated by anti-CCP2 status. No reference method or reference material exists for anti-PAD4; differences between methods are therefore reported as relative, and assay precision was not assessed. Receiver operating characteristic (ROC) curve analysis was used to evaluate the cutoff that yielded the best agreement between methods using Youden’s index. An UpSet plot was generated to visualize the intersections of positivity across all markers in the RA and control subgroups. Additionally, Spearman’s rank correlation plots were calculated to determine the association between markers.

Pairwise agreement between anti-PAD4 antibodies, RF IgM, RF IgA, and anti-CCP2 was measured by percent overall agreement (POA), percent positive agreement (PPA), and Cohen’s kappa, with 95% confidence intervals (CIs) derived using the asymptotic standard error method of Fleiss, Cohen, and Everitt.

Distributions of the continuous markers for each subgroup were illustrated in box-and-whisker plots, and pairwise comparisons were made between the RA group and each comparator group using Mann–Whitney U tests. ROC curve analysis was performed overall and separately for anti-CCP2-negative patients to evaluate the diagnostic accuracy of conventional RA biomarkers and anti-PAD4 antibodies in differentiating RA patients from the combined control group. Area under the curve (AUC) values with 95% CIs, estimated using 1000 bootstrap iterations, were used to quantify diagnostic performance.

Diagnostic performance of the markers for distinguishing RA patients from controls (AHV, SLE, PsA, and SjD patients) was measured by calculating sensitivity and specificity with the Wilson score method for 95% CIs and likelihood ratios (LRs) with the log method for CIs. Additionally, adjusted odds ratios (aORs) and profile-likelihood 95% CIs were estimated using Firth’s logistic regression with RA vs. controls as the binary outcome, adjusting for age and sex; subjects with missing age (*n* = 11) were excluded from adjusted models. *p*-values from the logistic regression models were adjusted for multiple comparisons using the Benjamini–Hochberg false discovery rate method. Adjusted *p*-values less than 0.05 were considered statistically significant. Diagnostic performance characteristics were calculated for the entire cohort and separately for anti-CCP2-negative patients.

Forest plots were created to show the aforementioned odds ratios (ORs) from Firth’s logistic regression for the association of each marker with RA diagnosis, adjusting for age and sex. To evaluate the association between anti-PAD4 marker positivity and treatment use in the RA cohort, Firth’s regression adjusted for age and sex was also used, and forest plots were created to display ORs of anti-PAD4 positivity as a predictor of RA treatment.

To assess the incremental diagnostic value of anti-PAD4 antibodies beyond conventional serology, Firth models containing age, sex, anti-CCP2, RF IgM, and RF IgA positivity were fitted with and without anti-PAD4 IgG (or IgA) positivity, in the entire cohort and in the anti-CCP2-negative subgroup. The independent association of anti-PAD4 with RA was tested with the penalized likelihood-ratio test, and the change in discrimination was quantified by the difference in AUC between model-predicted probabilities (DeLong test) and by the integrated discrimination improvement (IDI).

During the preparation of this manuscript, the authors used Claude (Anthropic) to assist with writing and debugging code (Python [version 3.14.8] and R [version 4.6.1]) used to generate figures, and to support language proofreading and checks of internal consistency (e.g., terminology, abbreviations, and formatting). The study’s conception, design, data analysis, interpretation, and all scientific conclusions are solely the work of the authors. The authors reviewed and verified all AI-assisted output and take full responsibility for the content of this publication.

## 3. Results

### 3.1. Cohort Characteristics

A total of 575 participants were included in this study, comprised of 192 RA patients (81 anti-CCP2 positive, 111 anti-CCP2 negative), 205 AHVs, 79 SLE, 64 PsA, and 35 SjD patients (Table 1). Most RA patients were female (81.2%), compared with 77.6% of AHVs, 89.9% of SLE, 60.9% of PsA, and 97.1% of SjD patients. RA patients’ mean age at visit was 56.5 years, similar to AHV (57.7), PsA (56.0), and SjD (60.0) patients, and higher than SLE patients (43.8). Serology positivity rates were higher for RA than the other subgroups: anti-PAD4 IgG (31.8% vs. 2.9% AHV, 13.9% SLE, 9.4% PsA, and 14.3% SjD), anti-PAD4 IgA (18.8% vs. 5.4% AHV, 3.8% SLE, 12.5% PsA, and 5.7% SjD), RF IgM (43.2% vs. 6.3% AHV, 10.1% SLE, 6.2% PsA, and 25.7% SjD), RF IgA (23.4% vs. 1.0% AHV, 6.3% SLE, 1.6% PsA, and 20.0% SjD), and anti-CCP2 (42.2% vs. 0.5% AHV, 1.3% SLE, 1.6% PsA, and 0.0% SjD). Serology levels were significantly higher in RA patients than in combined controls for RF IgM, RF IgA, and anti-CCP2.

When we zeroed in on the RA population, there was no statistically significant difference in age, sex, or ethnicity between the anti-CCP2-negative and anti-CCP2-positive groups. Similarly, there were no significant differences in disease duration or disease state (early, established, longstanding) between the RA groups. There were, however, significant differences between the two groups in CDAI scores, with the anti-CCP2-positive group having a larger proportion of patients in remission (30.0%) compared with the anti-CCP2-negative group (10.5%) and a smaller proportion of anti-CCP2-positive patients in the high-CDAI category (13.3%) than in the anti-CCP2-negative group (24.2%). Additionally, the following significant differences in serology positivity between anti-CCP2-positive and anti-CCP2-negative patients were found: anti-PAD4 IgG (50.6% vs. 18.0%), anti-PAD4 IgA (35.8% vs. 6.3%), RF IgM (76.5% vs. 18.9%), and RF IgA (45.7% vs. 7.2%). Serology levels were significantly higher for anti-CCP2-positive than anti-CCP2-negative patients for all markers analyzed (*p* < 0.001 for all markers).

### 3.2. Anti-PAD4 IgG Assays Demonstrate Correlation and Comparability

First, two independent methods were compared for the detection of anti-PAD4 IgG (PMAT and ELISA), and a positive correlation was found between the two tests (Spearman rho = 0.66, 95% CI: 0.55–0.74, Figure 1A). Passing–Bablok regression gave a slope of 0.83 (95% CI 0.71–0.92) and an intercept of −1.34 (95% CI −1.60 to −1.04), indicating a modest proportional difference (a 10-fold rise in PMAT corresponded to a ~6.8-fold rise in ELISA); the intercept reflects scale conversion between assays and not bias. Deming regression gave similar estimates (slope 0.82, 95% CI 0.74–0.91; intercept −1.29, 95% CI −1.51 to −1.06). Residuals showed mild deviation from linearity at low concentrations, and nine samples had ELISA values > 2 SD above prediction; excluding them did not materially change the fit (slope 0.81, 95% CI 0.70–0.89; intercept −1.30, 95% CI −1.53 to −1.01). At the respective cutoffs, 38 samples were ELISA-positive, and 29 PMAT-positive; 25 were positive by both, and 101 were negative by both (Figure 1A). PPA was 86.2% (95% CI 69.4–94.5), NPA 88.6% (81.5–93.2), POA 88.1% (81.8–92.4), and *kappa* = 0.67 (95% CI 0.53–0.81). Of 17 discordant samples, 13 were ELISA-positive/PMAT-negative, and 4 PMAT-positive/ELISA-negative (exact McNemar *p* = 0.049; continuity-corrected *p* = 0.052). Seven of the 13 were among the nine flagged samples; discordant samples were mainly RA (15/17). Rank correlation was weaker in anti-CCP2-negative (rho = 0.45, 95% CI 0.26–0.60; *n* = 86) than in anti-CCP2-positive samples (rho = 0.84, 95% CI 0.74–0.90; *n* = 57), possibly reflecting the narrower concentration range; qualitative agreement and regression estimates were similar between the groups.

In ROC curve analysis, an area under the curve (AUC) value of 0.87 was found (Figure 1B). A cutoff of ~937 median fluorescence intensity (MFI) yielded the highest agreement between methods (vs. 1000 MFI used by Martinez-Prat et al. [13]). In addition, the ELISA showed stronger correlation with classical RA markers such as anti-CCP2 (rho = 0.48 vs. 0.40), RF IgM (rho = 0.40 vs. 0.31), and RF IgA (rho = 0.47 vs. 0.35). At a cutoff of 1000 MFI, as used in the previous study, high levels of qualitative agreement were observed. Positive and negative percent agreements were 86.2% (95% CI = 69.4–94.5%) and 88.6% (95% CI = 81.5–93.2%), respectively. Kappa agreement was 0.670 (95% CI: 0.528–0.813).

### 3.3. Correlation Between RA Biomarkers

Next, we sought to analyze the correlation and marker combinations between anti-PAD4 antibodies and other RA biomarkers. An UpSet plot was used to visualize the intersections between the different RA autoantibodies (Figure 1D) for the entire cohort, including RA patients and controls. Most samples were negative by all methods (*n* = 373). Various combinations of biomarker positivity were observed, including 12 samples that were positive for all five biomarkers. The correlation between anti-PAD4 antibodies and conventional markers ranged from rho = 0.20 for anti-PAD4 IgA vs. anti-CCP2 to rho = 0.49 for RF IgA vs. anti-CCP2 (Figure 1E). All correlations were statistically significant. In the RA cohort, the associations showed similar trends but were more pronounced.

Pairwise agreement between anti-PAD4 antibodies and conventional RA markers was assessed across the entire cohort. Percent overall agreement (POA) was consistently high across all marker pairs, ranging from 79.1% to 88.2%, largely driven by the substantial number of double-negative samples, reflecting the relatively low positivity rate of anti-PAD4 antibodies in this cohort (Table 2). Among the anti-PAD4 comparisons, percent positive agreement (PPA) was substantially lower, ranging from 24.8% to 53.3%. Cohen’s kappa values were uniformly in the fair range (*kappa* = 0.22 to 0.39), consistent with the low prevalence of positivity driving the high POA. The highest agreement was observed between anti-PAD4 IgG and anti-PAD4 IgA (POA = 85.2%, PPA = 53.3%, k = 0.35), while the lowest PPA was seen between anti-PAD4 IgA and RF IgM (PPA = 24.8%, *kappa* = 0.22).

### 3.4. Distribution of Antibodies and Diagnostic Performance Among RA Patients and Controls

Anti-PAD4 antibodies were more widely distributed in RA patients than in any of the control groups, although the differences between the distributions were generally not statistically significant from the rest of the cohort (Figure 2A–E).

ROC curve analysis showed significant variation between the individual markers in their ability to discriminate RA from controls. The highest AUC was found for anti-CCP2 (AUC = 0.86), followed by the other markers in the following order: RF IgA (AUC = 0.69), RF IgM (AUC = 0.67), anti-PAD4 IgG (AUC = 0.55), and anti-PAD4 IgA (AUC = 0.52) (Figure 2F). When focusing on the anti-CCP2-negative subgroup, the AUCs diminished for all markers (Figure 2G).

In the entire cohort, anti-CCP2 demonstrated the highest diagnostic performance, with a sensitivity of 42.2% (95% CI: 35.4–49.3) and specificity of 99.2% (95% CI: 97.7–99.7), yielding the highest adjusted OR of 110.27 (95% CI: 37.39–536.31) and LR+ of 53.86 (95% CI: 17.24–168.28) (Table 3). RF IgM and RF IgA showed sensitivities of 43.2% and 23.4%, with specificities of 91.1% and 96.1% and adjusted ORs of 7.40 (95% CI: 4.75–11.75) and 7.14 (95% CI: 3.97–13.51), respectively. Anti-PAD4 IgG and IgA showed lower sensitivities of 31.8% and 18.8%, with specificities of 92.7% and 93.7% and adjusted ORs of 5.92 (95% CI: 3.65–9.84) and 3.48 (95% CI: 2.01–6.12), respectively. All markers achieved statistical significance after Benjamini–Hochberg correction (q < 0.001).

In the anti-CCP2-negative subgroup, anti-PAD4 IgG retained a statistically significant diagnostic association with RA (aOR = 2.87, 95% CI: 1.53–5.30, q = 0.005), while anti-PAD4 IgA did not reach significance (aOR = 1.06, 95% CI: 0.42–2.39, q = 0.888). RF IgM remained significant (aOR = 2.20, 95% CI: 1.19–3.95, q = 0.024), whereas RF IgA did not (aOR = 1.88, 95% CI: 0.76–4.38, q = 0.220). Notably, LR+ values in the anti-CCP2-negative subgroup were substantially attenuated compared to the full cohort, ranging from 1.01 to 2.46, reflecting the reduced discriminatory capacity of all markers in this population. The aforementioned adjusted ORs are illustrated in Figure 3A,B.

To assess whether anti-PAD4 IgG contributes diagnostic information beyond conventional serology, anti-PAD4 IgG positivity was added to a Firth model containing age, sex, anti-CCP2, RF IgM, and RF IgA positivity (Supplementary Table S1). Anti-PAD4 IgG remained independently associated with RA (aOR = 2.69, 95% CI: 1.43–5.04; penalized likelihood-ratio *p* = 0.003), whereas anti-PAD4 IgA did not (aOR = 1.00, *p* = 0.99). The gain in discrimination was small (ΔAUC = 0.021, 95% CI: −0.003 to 0.045; IDI = 0.014). In the anti-CCP2-negative subgroup, anti-PAD4 IgG retained an aOR of 2.69 (95% CI: 1.41–5.05, *p* = 0.003) after adjustment for RF IgM and RF IgA, with ΔAUC = 0.036 (95% CI: −0.005 to 0.078; IDI = 0.020). In these fully adjusted models, RF IgA was no longer associated with RA once RF IgM was included (aOR = 0.99 and 1.06 in the entire cohort and the anti-CCP2-negative subgroup, respectively).

Firth’s logistic regression models adjusted for age and sex were also used to evaluate the association between anti-PAD4 positivity and treatment used. Separate models estimated the odds of receiving each treatment among patients who were anti-PAD4 IgG positive vs. negative and among patients who were anti-PAD4 IgA positive vs. negative (Figure 3C). No statistically significant associations were observed between anti-PAD4 positivity and treatment use, with the exception of abatacept use among patients who were anti-PAD4 IgA positive, but this was diminished after Benjamini–Hochberg correction (OR = 10.96, 95% CI: 1.36–126.10, *p* = 0.04, q = 0.415).

### 3.5. Association with Clinical Parameters

When looking at the Spearman correlation between the markers and disease duration, none of the biomarkers showed an association with disease duration. Similarly, there was no significant correlation between any of the markers and age at diagnosis. Kruskal–Wallis testing initially suggested a borderline association between ethnicity and RF IgM across six ethnicity categories (χ^2^ = 11.14, df = 5, *p* = 0.05); however, three of these categories had inadequate sample sizes for reliable comparison (*n* = 1, *n* = 2, *n* = 2). Restricting the analysis to the three adequately sized groups (White, *n* = 165; Black, *n* = 14; Hispanic, *n* = 8) showed no significant association (χ^2^ = 5.06, df = 2, *p* = 0.080), consistent with the original borderline result being influenced by instability in the small groups rather than a genuine ethnicity effect. However, this remains underpowered to detect modest effects given the smaller sample sizes of the Black and Hispanic subgroups. Among the five markers tested for association with sex, RF IgA showed a significant difference between males and females (median 10.8 U/mL vs. 5.9 U/mL, Mann–Whitney U test, *p* = 0.025). However, this association dissipated after Benjamini–Hochberg correction for multiple comparisons (q = 0.118).

## 4. Discussion

The identification of anti-PAD4 antibodies in patients suspected of having RA offers promising avenues for improving diagnostic accuracy, particularly in seronegative cases [3,8,20,21]. Anti-PAD4 antibodies have been associated with more severe disease phenotypes and may serve as a valuable biomarker for patient stratification [14,15,16,20,22,23]. Their presence may help clinicians recognize RA at an earlier stage, potentially leading to timely intervention, treatment escalation, and improved outcomes [22].

In a recent meta-analysis [21], the pooled sensitivity and specificity for anti-PAD4 IgG were estimated to be 34% and 96%, respectively, against healthy individuals. In our study, the sensitivity was very similar (31.8%). The slightly lower value might be related to sample size and/or the higher percentage of anti-CCP2-negative patients.

Taken together, these data position anti-PAD4 IgG as a rule-in adjunct rather than a stand-alone diagnostic marker. Its positive likelihood ratio in anti-CCP2-negative patients (2.46) is comparable to that of RF IgM in the same population (2.13), and its association with RA persisted after adjustment for anti-CCP2, RF IgM, and RF IgA, indicating that anti-PAD4 IgG and RF identify partially distinct subsets of anti-CCP2-negative RA. The incremental gain in overall discrimination was small (ΔAUC 0.02–0.04), as expected for a marker with 18% sensitivity added to a panel that already contains anti-CCP2; its value lies in the anti-CCP2-negative population, in which conventional serology leaves the majority of patients unclassified. Anti-PAD4 IgA did not contribute independent diagnostic information in this cohort, and its interest lies in the isotype-level immunology and the exploratory treatment association rather than in diagnosis.

### 4.1. Method Comparison

Despite significant effort, standardization of autoantibody assays remains limited, which also applies to assays such as anti-CCP and RF [24]. In addition, until recently, no clinically validated assay was available for the detection of anti-PAD4 IgG. To translate findings from research studies into clinical practice, method comparison studies are important. Consequently, we compared ELISA with a PMAT assay used in several studies, including one of the largest cross-sectional studies of RA patients and controls. The two methods showed moderate-to-good agreement: rank correlation was rho = 0.66, qualitative agreement was substantial (*kappa* = 0.67), and regression indicated a modest proportional difference (Passing–Bablok slope 0.83, 95% CI 0.71–0.92), despite considerable differences in assay technology. Agreement was not uniform. Rank correlation was weaker in anti-CCP2-negative than in anti-CCP2-positive samples (rho = 0.45 vs. 0.84), possibly reflecting the narrower concentration range in the former. ELISA tended to classify more samples as positive than PMAT (13 ELISA-positive/PMAT-negative vs. 4 PMAT-positive/ELISA-negative samples; exact McNemar *p* = 0.049, continuity-corrected *p* = 0.052). Seven of the 13 ELISA-positive/PMAT-negative samples had ELISA values well above those predicted from PMAT (PMAT 80–653 MFI), whereas the remaining six had PMAT values of 420–973 MFI. The underlying reasons for the observed difference remain a matter of future research but might be attributed to differences in antigen exposure (epitope recognition), as the conjugate used to detect anti-PAD4 antibodies is a general difference in assay design. Elucidating such differences might enable future improvements in assays for the detection of anti-PAD4 antibodies. It has recently been demonstrated that certain epitopes of anti-PAD4 are recognized early during the course of disease and might serve as predictive factors for progression to clinically overt disease [25]. Even when the same antigen is used in different assays, differences in surface characteristics and coupling strategies might lead to different epitope exposure and, consequently, different results.

In addition, since the PAD4 enzyme exhibits auto-citrullination capabilities [26,27,28], the citrullination status of the PAD4 antigen used in different assays could potentially explain differences between assays [27]. However, it is important to mention that the study by Martinez-Prat et al. [13] used an early version of the assay and a prototype of the PMAT instrumentation. Consequently, further studies are needed once the final version of the PMAT anti-PAD4 assay becomes available.

### 4.2. Associations of Anti-PAD4 Antibodies of the IgA Isotype

Most studies on anti-PAD4 antibodies, with few exceptions [29,30], focused exclusively on IgG. Our study is one of the few to investigate the immune response of anti-PAD4 IgG and IgA. Along these lines, Gómez-Bañuelos et al. [29] also found significant differences between RA and controls for anti-PAD4 IgA. When comparing the correlation between anti-PAD4 IgG and IgA, a moderate association was reported by Gómez-Bañuelos et al. [29]. In our study, the correlation between the two anti-PAD4 isotypes was slightly lower compared to a previous study by Martinez-Prat et al. [30] (0.47 vs. 0.60), which was performed on 259 patient samples using the PMAT system.

Interestingly, in the study by Martinez-Prat et al. [30], the AUC comparing RA vs. controls was slightly higher for IgA compared to IgG (0.731 vs. 0.630), which was clearly different from the present study (AUC for IgG = 0.55 vs. AUC for IgA = 0.52). The reason for the different performance remains speculative, but it might be attributed to differences in the assay used to detect anti-PAD4 antibodies or, more specifically, related to the specificity of the secondary antibody used to detect IgA.

### 4.3. Associations of Biomarkers with Disease Duration, Disease Activity, and Joint Erosions

Anti-PAD4 antibodies have been reported to precede the clinical onset of RA; however, they arise after anti-CCP antibodies and RF [11,12], which is aligned with the theory that anti-PAD4 antibodies amplify rather than initiate the disease. In the meta-analysis by Zhang et al. [21], anti-PAD4-antibody-positive patients had slightly longer disease duration (1.22 years), but heterogeneity in the data from the different studies was reported. Similarly, no association between disease duration and anti-PAD4 levels was observed in the present study (Spearman rho = 0.06 for ELISA and 0.10 for PMAT, both non-significant).

While some previous studies reported an association between anti-PAD4 antibodies and disease activity, a clear consensus for such an association is still pending [20,21]. The current study did not find a statistical difference based on anti-PAD4 antibodies. A possible explanation is the broad spectrum of anti-PAD4 antibodies, which differ significantly in terms of their effect on PAD enzyme activity. While some anti-PAD4 antibodies activate enzyme function, other antibody specificities exhibit inactivating properties [16,17,18]. This concept is currently being explored as a potential therapy using a bispecific antibody that targets both PAD2 and PAD4 [31].

The association between anti-PAD4 IgA and abatacept treatment did not survive correction for multiple testing. Given the extremely small sample size in this subgroup (*n* = 3) and the resulting wide confidence interval (1.36–126.10), this result should be interpreted as exploratory only. It is nonetheless directionally consistent with previous studies. Darrah et al. showed an association between anti-PAD4 IgG and treatment escalation [22]. In addition, the findings from Martinez-Prat et al. indicate an association between anti-PAD4 IgA and biologic use. In this context, it is noteworthy that abatacept belongs to the group of biologics but is often only used if first-line biologics such as anti-tumor necrosis factor (TNF) agents fail and treatment is escalated. This association between anti-PAD4 IgA and abatacept treatment warrants confirmation in a larger, adequately powered sample.

### 4.4. Strengths and Limitations of the Study

This is the first study to compare the clinical performance of anti-PAD4 (IgA, IgG) together with classical RA biomarkers (anti-CCP2, RF IgA, and RF IgM) in a CLIA laboratory setting using a fully validated LDT in a cohort of well-characterized RA patients and controls. It also uniquely compares two independent methods for the detection of anti-PAD4 IgG, namely ELISA and PMAT.

Although our RA cohort was well characterized, there are some limitations in terms of clinical data. First, our dataset did not include specific data on joint erosions, such as Sharp–van der Heijde scores. Furthermore, no data on radiographic progression were available. In addition, the treatment data were limited, as they did not include information on treatment initiation or duration. Lastly, clinical variables (disease duration, disease stage, and CDAI) were available only for RA patients, so the incremental analyses could adjust for age and sex but not for the full clinical presentation; evaluation of anti-PAD4 against the complete 2010 ACR/EULAR classification score would require a prospective cohort of patients presenting with undifferentiated arthritis.

## 5. Conclusions

Our study provides further data and evidence for the presence of anti-PAD4 antibodies in RA patients and provides further insight into their interplay with classical RA biomarkers, namely anti-CCP2, RF IgM, and RF IgA. In addition, our data provide novel insights into the impact of the detection method on the diagnostic performance and disease associations of anti-PAD4 antibodies. Anti-PAD4 IgG provides modest diagnostic information that is independent of anti-CCP2 and RF and is most relevant in anti-CCP2-negative patients.

## Figures and Tables

**Figure 1:**
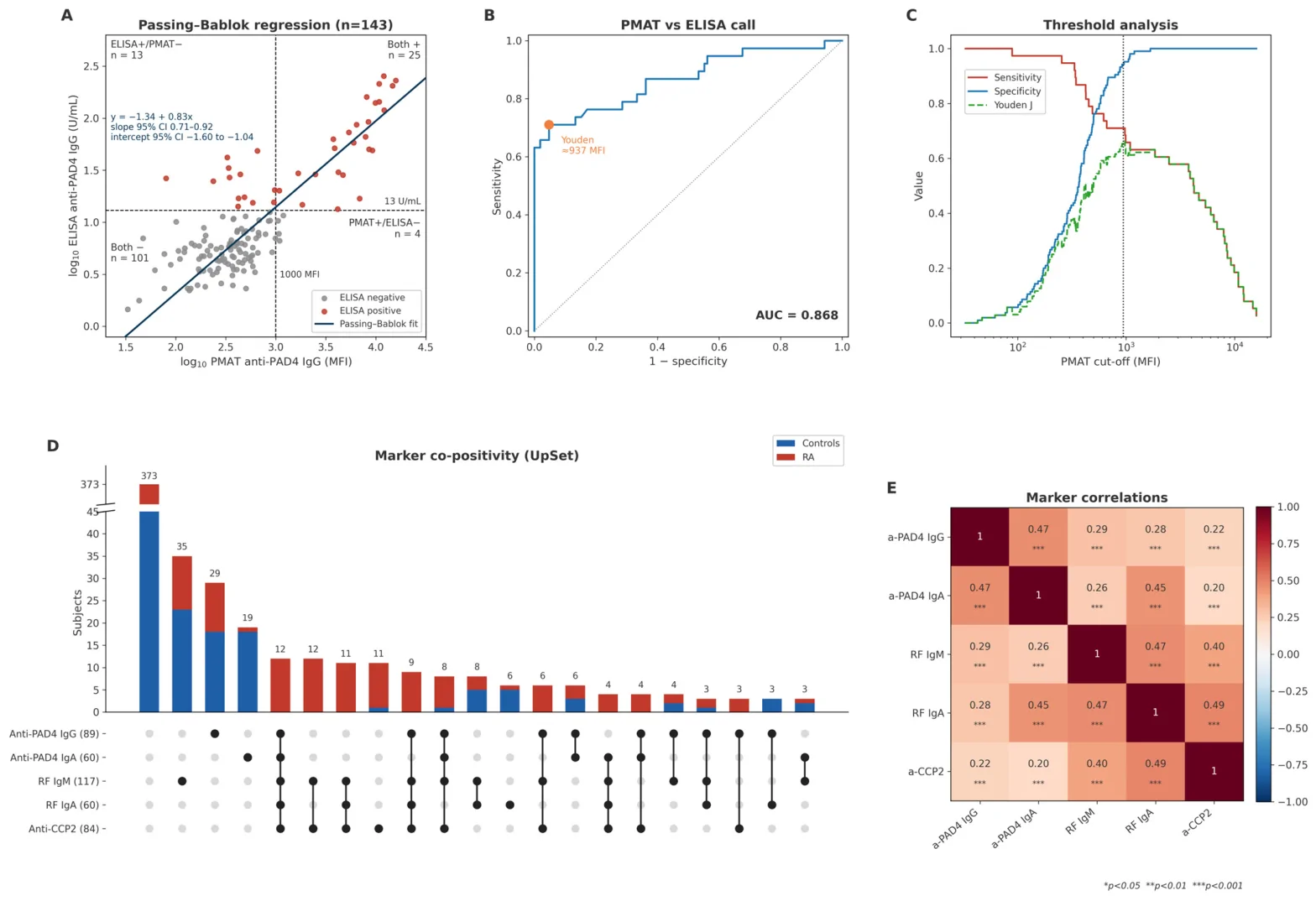
Anti-PAD4 assay comparison and RA-biomarker co-positivity. (**A**–**C**) Comparison of anti-PAD4 IgG measured by particle-based multi-analyte technology (PMAT) vs. ELISA, in the subset of samples run on both platforms (*n* = 143), shown on log10 axes. Dashed lines mark the assay cutoffs (1000 MFI; 13 U/mL), and counts are given for each agreement category (PMAT as comparator). The solid line is the Passing–Bablok fit (log10 ELISA = −1.34 + 0.83 × log10 PMAT). (**A**) Correlation of PMAT (median fluorescence intensity, MFI) and ELISA (U/mL) concentrations on log–log axes; points are colored by the ELISA positivity call, and the dashed line is the least-squares fit (Spearman rho = 0.658, 95% CI: 0.553–0.742). (**B**) Receiver operating characteristic curve for PMAT classifying the ELISA-positive call, i.e., agreement between assays rather than diagnosis of RA, with area under the curve (AUC) = 0.868; the orange point marks the Youden-optimal threshold (≈937 MFI), and the dotted diagonal denotes no discrimination. (**C**) Sensitivity, specificity, and Youden J of PMAT relative to the ELISA call across PMAT cutoffs; the dotted vertical line marks the Youden-optimal cutoff. (**D**,**E**) Co-positivity of five RA biomarkers (anti-PAD4 IgG, anti-PAD4 IgA, RF IgM, RF IgA, and anti-CCP2) across the full cohort (*n* = 575). (**D**) UpSet plot: bars show the size of each positivity combination, stacked by group (RA, red; controls, blue); filled dots below indicate the markers defining each combination, with per-marker positivity totals in parentheses. For visual purposes, only intersections with at least *n* = 3 patients are shown. (**E**) Pairwise Spearman correlations between the five marker concentrations; asterisks indicate significance (* *p* < 0.05, ** *p* < 0.01, *** *p* < 0.001).

**Figure 2:**
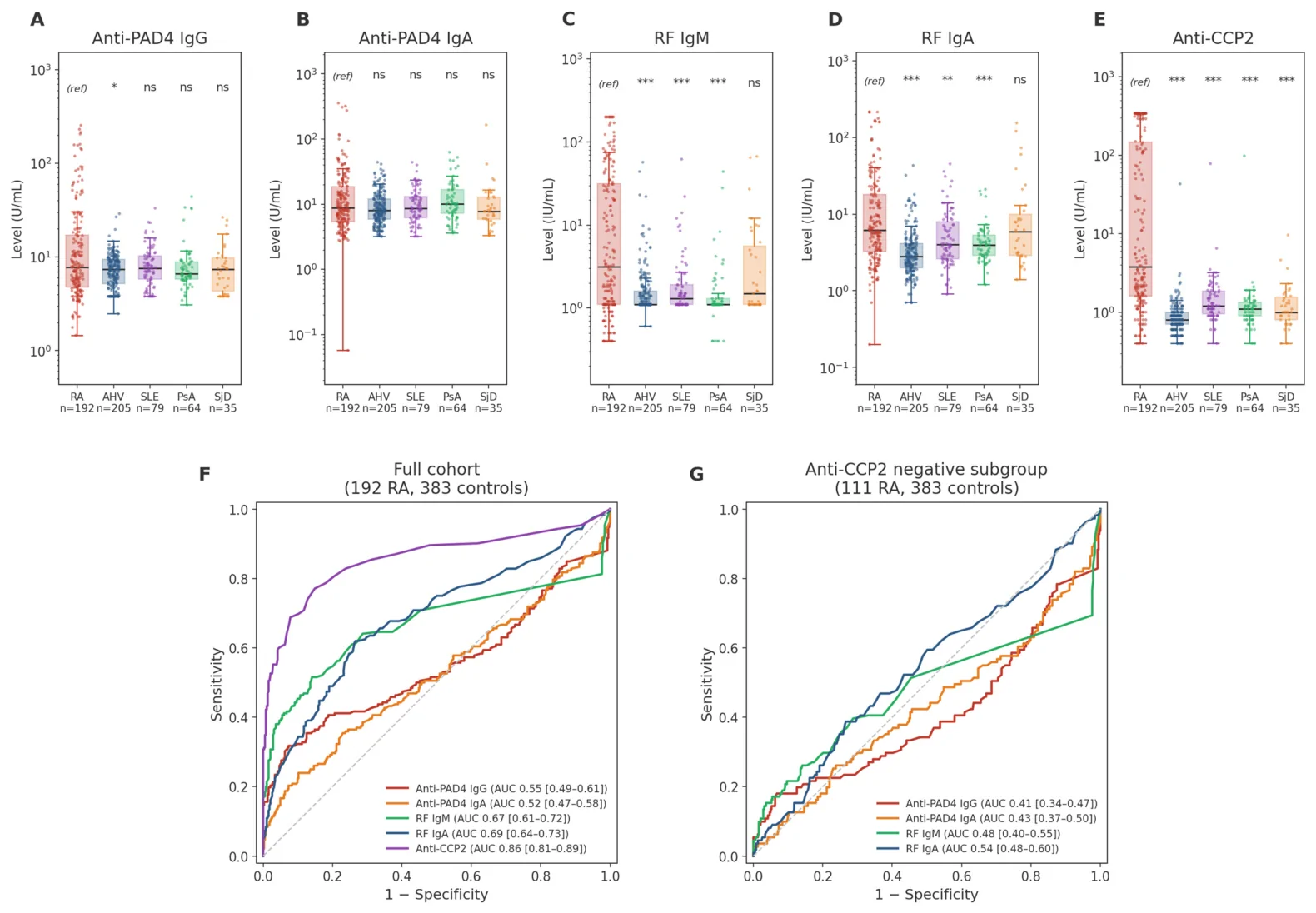
Biomarker concentrations by diagnostic group and diagnostic performance for RA. (**A**–**E**) Serum concentrations of five RA biomarkers across diagnostic groups: (**A**) anti-PAD4 IgG, (**B**) anti-PAD4 IgA, (**C**) RF IgM, (**D**) RF IgA, and (**E**) anti-CCP2. Each panel shows box plots (median, interquartile range, and 1.5 × IQR whiskers) with jittered individual points on a log scale; group sizes are given beneath each label. Asterisks denote pairwise comparisons of each control group against RA (reference) by the Mann–Whitney U test (* *p* < 0.05, ** *p* < 0.01, *** *p* < 0.001; ns, not significant). (**F**,**G**) Receiver operating characteristic curves for each marker discriminating RA from controls, using continuous marker concentrations. (**F**) Full cohort (192 RA, 383 controls), all five markers. (**G**) Anti-CCP2-negative subgroup (111 RA, 383 controls); anti-CCP2 is omitted because it defines the stratum. Legends report the area under the curve (AUC) with 95% confidence intervals from 1000 bootstrap resamples, and the dotted diagonal denotes no discrimination.

**Figure 3:**
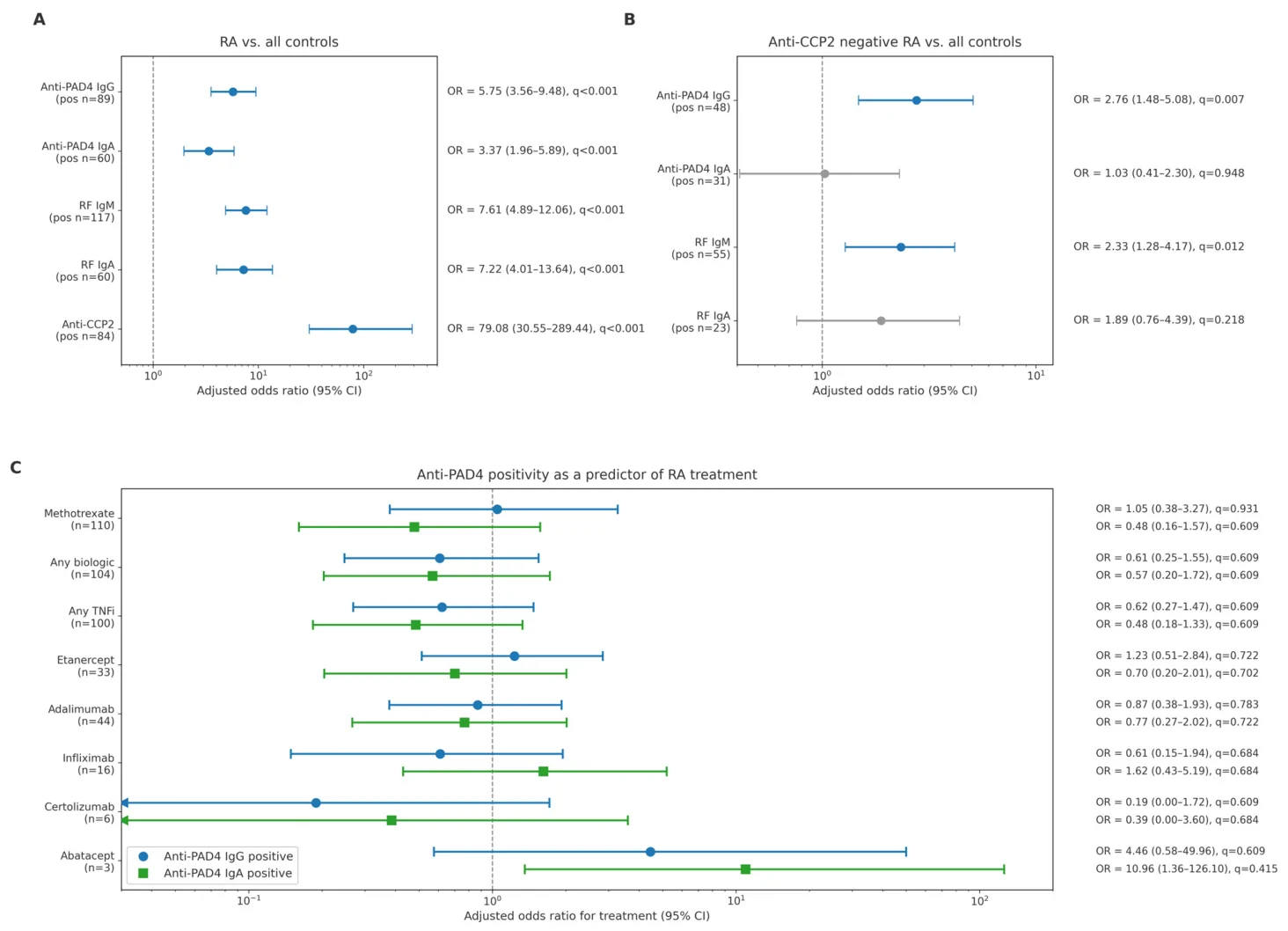
Adjusted associations of RA biomarkers with diagnosis and of anti-PAD4 positivity with treatment. Forest plots of adjusted odds ratios (ORs) for RA diagnosis. Each marker’s positivity was entered as the predictor in a separate Firth penalized logistic regression, adjusted for age and sex, with profile-likelihood 95% confidence intervals. (**A**) RA vs. all controls (190 RA, 374 controls with complete age data); all five markers are shown. (**B**) Anti-CCP2-negative RA vs. all controls (109 RA, 374 controls); anti-CCP2 is omitted because it defines the stratum. Points are colored blue where the interval excludes 1 and grey where it does not; y-axis labels give the number of marker-positive subjects in each cohort. The dashed vertical line marks OR = 1. (**C**) Forest plot of anti-PAD4 positivity as a predictor of being on each treatment, among RA patients only. For each treatment, anti-PAD4 IgG positivity (blue circles) and anti-PAD4 IgA positivity (green squares) were entered as predictors in separate Firth models adjusted for age and sex, with profile-likelihood 95% confidence intervals; y-axis labels give the number of RA patients on each treatment. Arrowheads indicate intervals extending beyond the axis range. Any TNFi denotes any of etanercept, adalimumab, infliximab, certolizumab, or golimumab; any biologic denotes any TNFi, abatacept, or tocilizumab; golimumab and tocilizumab (*n* = 1 each) were not estimable and are not shown.

**Table 1:** Cohort characteristics.

	All Cohorts							RA Cohort		
Characteristic	RA (*n* = 192)	AHV (*n* = 205)	SLE (*n* = 79)	PsA (*n* = 64)	SjD (*n* = 35)	All Controls (*n* = 383)	*p*-Value	Anti-CCP2+ (*n* = 81)	Anti-CCP2− (*n* = 111)	*p*-Value
Demographics										
Age (years), mean ± SD	56.5 ± 13.3	57.7 ± 15.2	43.8 ± 10.2	56.0 ± 16.8	60.0 ± 9.5	54.7 ± 15.2	0.349	57.8 ± 14.4	55.6 ± 12.5	0.099
Female sex, *n* (%)	156 (81.2%)	159 (77.6%)	71 (89.9%)	39 (60.9%)	34 (97.1%)	303 (79.1%)	0.547	62 (76.5%)	94 (84.7%)	0.153
Ethnicity (RA), *n* (%)										
White	165 (85.9%)	—	—	—	—	—	—	68 (84.0%)	97 (87.4%)	0.683
Black	14 (7.3%)	—	—	—	—	—	—	6 (7.4%)	8 (7.2%)	
Hispanic	8 (4.2%)	—	—	—	—	—	—	5 (6.2%)	3 (2.7%)	
Other/Not Disclosed	5 (2.6%)	—	—	—	—	—	—	2 (2.5%)	3 (2.7%)	
Disease Characteristics (RA)										
Disease duration, years, median (IQR)	7.0 (3.0–14.0)	—	—	—	—	—	—	7.0 (3.0–17.2)	6.0 (4.0–13.0)	0.772
Disease state, *n* (%)	—	—	—	—	—	—	—	—	—	0.125
Early	33 (17.5%)	—	—	—	—	—	—	18 (22.5%)	15 (13.8%)	
Established	93 (49.2%)	—	—	—	—	—	—	33 (41.2%)	60 (55.0%)	
Longstanding	63 (33.3%)	—	—	—	—	—	—	29 (36.2%)	34 (31.2%)	
CDAI category, *n* (%)	—	—	—	—	—	—	—	—	—	0.011
Remission	28 (18.1%)	—	—	—	—	—	—	18 (30.0%)	10 (10.5%)	
Low	59 (38.1%)	—	—	—	—	—	—	23 (38.3%)	36 (37.9%)	
Moderate	37 (23.9%)	—	—	—	—	—	—	11 (18.3%)	26 (27.4%)	
High	31 (20.0%)	—	—	—	—	—	—	8 (13.3%)	23 (24.2%)	
Serology—Positivity, *n* (%)										
Anti-PAD4 IgG	61 (31.8%)	6 (2.9%)	11 (13.9%)	6 (9.4%)	5 (14.3%)	28 (7.3%)	<0.001	41 (50.6%)	20 (18.0%)	<0.001
Anti-PAD4 IgA	36 (18.8%)	11 (5.4%)	3 (3.8%)	8 (12.5%)	2 (5.7%)	24 (6.3%)	<0.001	29 (35.8%)	7 (6.3%)	<0.001
RF IgM	83 (43.2%)	13 (6.3%)	8 (10.1%)	4 (6.2%)	9 (25.7%)	34 (8.9%)	<0.001	62 (76.5%)	21 (18.9%)	<0.001
RF IgA	45 (23.4%)	2 (1.0%)	5 (6.3%)	1 (1.6%)	7 (20.0%)	15 (3.9%)	<0.001	37 (45.7%)	8 (7.2%)	<0.001
Anti-CCP2	81 (42.2%)	1 (0.5%)	1 (1.3%)	1 (1.6%)	0 (0.0%)	3 (0.8%)	<0.001	81 (100.0%)	0 (0.0%)	*
Serology—Antibody Levels, Median (IQR)										
Anti-PAD4 IgG, U/mL	7.7 (4.8–17.1)	7.4 (5.2–9.4)	7.6 (5.8–10.3)	6.6 (5.8–8.9)	7.4 (4.3–9.8)	7.4 (5.4–9.4)	0.056	13.0 (7.5–48.7)	5.9 (4.1–8.9)	<0.001
Anti-PAD4 IgA, U/mL	8.8 (5.4–18.8)	8.0 (5.9–12.1)	8.7 (6.2–13.2)	10.1 (7.2–16.7)	7.8 (5.9–12.8)	8.5 (6.0–13.1)	0.362	13.2 (7.2–32.9)	7.6 (4.5–13.5)	<0.001
RF IgM, IU/mL	3.2 (1.1–31.5)	1.1 (1.1–1.6)	1.3 (1.1–1.9)	1.1 (1.1–1.3)	1.5 (1.1–5.6)	1.1 (1.1–1.8)	<0.001	31.0 (7.6–89.0)	1.2 (0.9–3.0)	<0.001
RF IgA, IU/mL	6.2 (3.2–18.0)	2.8 (2.0–4.1)	4.0 (2.6–7.9)	4.0 (2.9–5.3)	5.9 (2.9–9.9)	3.3 (2.3–5.2)	<0.001	17.0 (7.5–39.0)	3.7 (2.3–6.5)	<0.001
Anti-CCP2, U/mL	3.8 (1.6–146.2)	0.8 (0.7–1.0)	1.2 (0.9–1.9)	1.1 (0.9–1.3)	1.0 (0.8–1.6)	0.9 (0.7–1.2)	<0.001	291.0 (74.0–340.0)	1.7 (1.1–2.8)	<0.001

*p*-value column compares RA vs. all controls combined throughout: continuous variables (age, antibody levels) by Mann–Whitney U test; categorical variables (sex, serology positivity) by Pearson’s χ^2^ test. Ethnicity and disease-characteristic rows are reported within RA only (— for other groups). Anti-CCP2+/Anti-CCP2− column: uncorrected Pearson’s χ^2^ test (categorical variables) or Mann–Whitney U test (continuous variables), within RA. * Anti-CCP2 positivity defines the Anti-CCP2+/Anti-CCP2− strata, so values are 100%/0% by construction, and no test is performed. Disease characteristics available in RA patients with complete records: disease duration *n* = 189, disease state *n* = 189, CDAI category *n* = 155. AHV, apparently healthy volunteer; CDAI, Clinical Disease Activity Index; IQR, interquartile range; PAD4, peptidylarginine deiminase 4; RF, rheumatoid factor. Bold *p*-values indicate *p* < 0.05.

**Table 2:** Pairwise agreement between RA biomarkers across the full cohort (*n* = 575).

Marker 1	Marker 2	*n*	TP	FP	FN	TN	POA (95% CI)	PPA (95% CI)	κ (95% CI)
Anti-PAD4 IgG	Anti-PAD4 IgA	575	32	57	28	458	85.2% (82.1–87.9)	53.3% (40.9–65.4)	0.35 (0.24–0.46)
Anti-PAD4 IgG	RF IgM	575	43	46	74	412	79.1% (75.6–82.3)	36.8% (28.6–45.8)	0.29 (0.20–0.39)
Anti-PAD4 IgG	RF IgA	575	29	60	31	455	84.2% (81.0–86.9)	48.3% (36.2–60.7)	0.30 (0.19–0.41)
Anti-PAD4 IgG	Anti-CCP2	575	42	47	42	444	84.5% (81.3–87.2)	50.0% (39.5–60.5)	0.39 (0.29–0.50)
Anti-PAD4 IgA	RF IgM	575	29	31	88	427	79.3% (75.8–82.4)	24.8% (17.8–33.3)	0.22 (0.12–0.32)
Anti-PAD4 IgA	RF IgA	575	18	42	42	473	85.4% (82.3–88.0)	30.0% (19.9–42.5)	0.22 (0.10–0.33)
Anti-PAD4 IgA	Anti-CCP2	575	30	30	54	461	85.4% (82.3–88.0)	35.7% (26.3–46.4)	0.34 (0.23–0.45)
**RF IgM**	**RF IgA**	**575**	**48**	**69**	**12**	**446**	**85.9% (82.8–88.5)**	**80.0% (68.2–88.2)**	**0.47 (0.37–0.56)**
**RF IgM**	**Anti-CCP2**	**575**	**63**	**54**	**21**	**437**	**87.0% (84.0–89.5)**	**75.0% (64.8–83.0)**	**0.55 (0.46–0.64)**
**RF IgA**	**Anti-CCP2**	**575**	**38**	**22**	**46**	**469**	**88.2% (85.3–90.6)**	**45.2% (35.0–55.9)**	**0.46 (0.35–0.57)**

TP, both markers positive; FP, marker 1 positive, marker 2 negative; FN, marker 1 negative, marker 2 positive; TN, both negative. POA, percent overall agreement = (TP + TN)/*n*; PPA, positive percent agreement = TP/(TP + FN); both with 95% Wilson score CIs. κ, Cohen’s kappa (95% CI, asymptotic Fleiss–Cohen–Everitt variance). Kappa interpretation: <0.20 slight; 0.20–0.39 fair; 0.40–0.59 moderate; 0.60–0.79 substantial; ≥0.80 almost perfect. Bold κ indicates moderate or higher agreement (κ ≥ 0.40). *n* = 575 for all pairs.

**Table 3:** Diagnostic performance of biomarkers for rheumatoid arthritis vs. all controls.

Marker	RA Pos/ Total	Controls Pos/ Total	Sensitivity % (95% CI)	Specificity % (95% CI)	LR+ (95% CI)	LR− (95% CI)	Adj. OR (95% CI) †	q (BH) ‡
**Entire Cohort**								
Anti-PAD4 IgG	61/192	28/383	31.8 (25.6–38.7)	92.7 (89.6–94.9)	4.35 (2.86–6.56)	0.74 (0.67–0.81)	5.92 (3.65–9.84)	**<0.001**
Anti-PAD4 IgA	36/192	24/383	18.8 (13.9–24.9)	93.7 (90.8–95.8)	2.99 (1.84–4.87)	0.87 (0.81–0.93)	3.48 (2.01–6.12)	**<0.001**
RF IgM	83/192	34/383	43.2 (36.4–50.3)	91.1 (87.9–93.6)	4.87 (3.40–6.98)	0.62 (0.55–0.71)	7.40 (4.75–11.75)	**<0.001**
RF IgA	45/192	15/383	23.4 (18.0–29.9)	96.1 (93.6–97.6)	5.98 (3.42–10.46)	0.80 (0.73–0.86)	7.14 (3.97–13.51)	**<0.001**
Anti-CCP2	81/192	3/383	42.2 (35.4–49.3)	99.2 (97.7–99.7)	53.86 (17.24–168.28)	0.58 (0.52–0.66)	110.27 (37.39–536.31)	**<0.001**
**Anti-CCP2 Negative**								
Anti-PAD4 IgG	20/111	28/383	18.0 (12.0–26.2)	92.7 (89.6–94.9)	2.46 (1.45–4.20)	0.88 (0.81–0.97)	2.87 (1.53–5.30)	**0.005**
Anti-PAD4 IgA	7/111	24/383	6.3 (3.1–12.4)	93.7 (90.8–95.8)	1.01 (0.45–2.27)	1.00 (0.95–1.06)	1.06 (0.42–2.39)	**0.888**
RF IgM	21/111	34/383	18.9 (12.7–27.2)	91.1 (87.9–93.6)	2.13 (1.29–3.52)	0.89 (0.81–0.98)	2.20 (1.19–3.95)	**0.024**
RF IgA	8/111	15/383	7.2 (3.7–13.6)	96.1 (93.6–97.6)	1.84 (0.80–4.23)	0.97 (0.91–1.02)	1.88 (0.76–4.38)	**0.220**

Sensitivity and specificity use the binary positivity threshold for each marker; 95% CIs by the Wilson score method. LR+ and LR− with 95% CIs by the log method. RA *n* = 192; controls *n* = 383. In the anti-CCP2-negative subgroup, RA *n* = 111 and controls *n* = 383. Adjusted models exclude 11 subjects with missing age (entire cohort: 190 RA, 374 controls; anti-CCP2-negative subgroup: 109 RA, 374 controls). † Adjusted odds ratio (RA vs. controls) from Firth penalized logistic regression, adjusted for age and sex, with profile-likelihood 95% CIs. ‡ q (BH): raw penalized-likelihood-ratio *p*-values corrected for multiple comparisons by the Benjamini–Hochberg procedure, controlling the false discovery rate within each panel separately (5 tests, entire cohort; 4 tests, anti-CCP2-negative subgroup). Bold indicates q < 0.05. Anti-PAD4 IgG and RF IgM remain significant in the anti-CCP2-negative subgroup after correction.

## Data Availability

The data supporting the reported results is stored within Exagen’s database and can be obtained per request and application.

## Supplementary Materials

The following supporting information can be downloaded at: https://www.mdpi.com/article/10.3390/diagnostics16213615/s1, Table S1. Incremental diagnostic value of anti-PAD4 antibodies beyond conventional serology.

## Abbreviations

The following abbreviations are used in this manuscript:

ACPA	Anti-citrullinated protein/peptide antibody
ACR	American College of Rheumatology
aOR	Adjusted odds ratio
ARD	Autoimmune rheumatic disease
AHV	Apparently healthy volunteer
AUC	Area under the curve
CCP2	Cyclic citrullinated peptide 2
CDAI	Clinical Disease Activity Index
CI	Confidence interval
DAS28	Disease Activity Score in 28 joints
DMARD	Disease-modifying antirheumatic drug
ELISA	Enzyme-linked immunosorbent assay
IDI	Integrated discrimination improvement
IQR	Interquartile range
LDT	Laboratory developed test
LR	Likelihood ratio
MFI	Median fluorescence intensity
NPA	Negative percent agreement
OR	Odds ratio
PAD4	Peptidylarginine deiminase 4
PMAT	Particle-based multi-analyte technology
POA	Percent overall agreement
PPA	Percent positive agreement
PsA	Psoriatic arthritis
q (BH)	Benjamini–Hochberg adjusted *p*-value
RA	Rheumatoid arthritis
RF	Rheumatoid factor
ROC	Receiver operating characteristic
SjD	Sjögren’s disease
SLE	Systemic lupus erythematosus
TNF	Tumor necrosis factor
