# Supplementary material for: Diagnostic and Clinical Utility of Novel Anti-PAD4 Antibodies in Anti-CCP2-Negative and Anti-CCP2-Positive Rheumatoid Arthritis
Source: Diagnostics (Basel). 2026 Nov 1;16(21):3615. doi: 10.3390/diagnostics16213615 (PMC13761568; doi:10.3390/diagnostics16213615)
Supplement: Supplementary file 1 [file diagnostics-16-03615-s001.zip › diagnostics-4601665-supplementary.pdf]

Supplementary Table S1. Incremental diagnostic value of anti-PAD4 antibodies beyond conventional serology.

Model	RA / controls (n)	Marker added	aOR (95% CI)†	PLR p‡	AUC base	AUC + marker	ΔAUC (95% CI)§	IDI
Entire cohort	190 / 374	Anti-PAD4 IgG	2.69 (1.43–5.04)	0.003	0.752	0.772	0.021 (−0.003 to 0.045)	0.014
Entire cohort	190 / 374	Anti-PAD4 IgA	1.00 (0.41–2.23)	0.99	0.752	0.752	0.000 (0.000 to 0.000)	−0.001
Anti-CCP2-negative RA vs. all controls	109 / 374	Anti-PAD4 IgG	2.69 (1.41–5.05)	0.003	0.569	0.605	0.036 (−0.005 to 0.078)	0.020
Anti-CCP2-negative RA vs. all controls	109 / 374	Anti-PAD4 IgA	1.01 (0.40–2.28)	0.99	0.569	0.569	0.000 (−0.001 to 0.001)	0.000

Base model: Firth penalized logistic regression for RA vs. controls with age, sex, anti-CCP2, RF IgM, and RF IgA positivity; in the anti-CCP2-negative panel, anti-CCP2 is omitted because it defines the stratum. Subjects with missing age (2 RA, 9 controls) were excluded. † Adjusted odds ratio for the added marker in the full model, with profile-likelihood 95% CI. ‡ Penalized likelihood-ratio test for the added marker. § Difference in AUC between model-predicted probabilities of the full and base models, with DeLong 95% CI; IDI, integrated discrimination improvement. In the full models, adjusted ORs for the conventional markers were: entire cohort — anti-CCP2 53.6, RF IgM 2.18, RF IgA 0.99; anti-CCP2-negative subgroup — RF IgM 2.03, RF IgA 1.06.
